# Apoptosis-Inducing Factor, Protein Expression, and Apoptosis Changes with Glutamine in Podocytes Cells Exposed with Cisplatin

**DOI:** 10.1155/2021/5599452

**Published:** 2021-04-21

**Authors:** Imam Susilo, Himmayatussorofil Maulida, Lindawati Alimsardjono, Dyah Fauziah, Herinda Pertiwi

**Affiliations:** ^1^Department of Pathological Anatomy, Universitas Airlangga, Jalan Mayjen Prof. Dr. Moestopo 47, Surabaya, Indonesia; ^2^Medicine Undergraduate Program, Universitas Airlangga, Jalan Mayjen Prof. Dr. Moestopo 47, Surabaya, Indonesia; ^3^Department of Microbiology, Universitas Airlangga, Jalan Mayjen Prof. Dr. Moestopo 47, Surabaya, Indonesia; ^4^Department of Health Studies, Universitas Airlangga, Jalan Dharmawangsa Dalam 28-30, Surabaya, Indonesia

## Abstract

Cisplatin is a well-known chemotherapeutic drug. It is one of the most effective anticancer agents and is widely used for the treatment of several types of tumors. However, side effects in normal tissues and organs, such as nephrotoxicity that induces apoptosis in epithelial cells in the kidney, limit the use of cisplatin. Glutamine is a substrate for the synthesis of glutathione as an antioxidant and promotes HSP70 release, protecting cells from apoptosis induced by different stimuli. In the present study, we investigated the protective effect of glutamine on cisplatin nephrotoxicity in the kidney. Mice were divided into three groups such as a group of control (P0), a group of intraperitoneal injection of a single dose cisplatin 20 mg/kg BW at 7th day (P1), and a group of intravenous glutamine injection 100 mg/kg BW at days 1–7 and given an intraperitoneal injection of single dose cisplatin 20 mg/kg BW at 7th day (P2). Measurement of AIF expression and apoptotic cells was carried out by immunohistochemical methods. The number of AIF expressions and apoptotic cells is expressed in the Allred score. AIF expression result is as follows: P0: 3.29 ± 0.79, P1: 5.32 ± 0.68, and P2: 4.49 ± 0.47. Apoptosis result is as follows: P0: 3.04 ± 0.70, P1: 5.26 ± 0.53, and P2: 4.44 ± 0.41. There is a decreased expression of AIF on intravenous glutamine administration, followed by a decrease in apoptosis in the podocyte. In conclusion, glutamine administration might represent the treatment of nephrotoxic-induced cisplatin.

## 1. Introduction

Cisplatin (CAS No.15663-27-1, MF-Cl2H6N2Pt; NCF-119875) or cisplatinum, also called cis-diamminedichloroplatinum (II), is the first platinum drug approved globally for the treatment of cancer in 1978. Cisplatin is one of the most effective anticancer agents and is widely used for the treatment of several types of tumors. Cisplatin can bind to purine bases in DNA, interfere with DNA repair, cause DNA damage, and then induce apoptosis in cancer cells [1, 2]. Behind the benefits of cisplatin, there are various side effects, and one of them is nephrotoxic. The mechanisms of cisplatin-induced nephrotoxicity are complex and involve many cellular processes including oxidative stress, apoptosis, and inflammation [3, 4]. Tsuruya et al. in 2003 observed that renal epithelial cells deficient in TNFR1 and Fas were resistant to cisplatin-induced cell death [5]. Seth et al. in 2005 identified that cisplatin increases caspase-2 and caspase-3 activities [6]. Takeda et al. in 1997 demonstrated that caspase-8 inhibition reduced cisplatin-induced cell death in vitro [7].

Apoptosis-inducing factor (AIF) is a mitochondrial oxidoreductase that plays a role in oxidative phosphorylation and redox control in normal cells. AIF was initially cloned and identified as a caspase-independent mitochondrial effector of apoptotic cell death. AIF is usually confined to the mitochondrial intermembrane space and released in response to death stimuli. In the cytoplasm, AIF can promote apoptosis by interacting with CYP, in which CypA assists in apoptogenic cytonuclear translocation of AIF. In contrast, Hsp70 maintains AIF in the cytoplasm and, therefore, can delay or prevent initiation of nuclear apoptosis. In the nucleus, AIF affects the chromosome condensation and fragmentation. Besides, AIF can induce mitochondria to release apoptogenic-proteins including cytochrome c and caspase-9 [8–10].

Glutamine is an *α*-amino acid and is the most abundant free amino acid in the body. [11]. Glutamine plays an important role in the modulation of HSP expression via the biosynthetic pathway hexosamine (HBP). HSP70 (HSP72 and HSP 73) acts as antiapoptosis protein [12, 13]. Glutamine (via glutamate), cysteine, and glycine are precursor amino acids for the synthesis of GSH. GSH is an antioxidant that can react directly with ROS and produce oxidized GSH (GSSG) [11].

Cell damage due to exposure to cisplatin chemotherapy is generally studied in the proximal renal tubule area because there is a process of reabsorption and primary urine secretion in that area. However, kidney cell damage may occur in glomerular visceral epithelial cells (podocyte) as a filtration site in the urinary system, which causes all substances to pass through the filtering process in the glomerulus before going to the proximal tubule and directly exposed to cisplatin. Therefore, research on apoptosis and AIF expression was carried out on glomerular visceral epithelial cells (podocyte). This study is analyzing the nephroprotective effect of intravenous glutamine on the incidence of apoptosis of the glomerular epithelial cells (podocyte) by examining the expression of AIF which is the initiator of apoptosis in the apoptosis-independent caspase. This research is expected to be an alternative problem solving for kidney failure caused by cisplatin chemotherapy modalities.

## 2. Materials and Methods

### 2.1. Animal and Housing

2-3 months old Wistar male white mice weighing 150–200 g were used after one week for proper acclimatization to the animal house conditions (12 h lighting cycle and 29–31°C temperature) with free access to water and standard rodent chow. All experimental procedures were conducted according to the ethical standards approved by the Institutional Animal Ethics Committee guidelines for animal care and use, Airlangga University, Indonesia. Animals were randomly divided into three groups with 10 animals in each group. The first group was treated as the control group (P0). The second group was treated with cisplatin (20 mg/kg I.p) as a positive control (P1). The third group was injected with glutamine (100 mg/kg, i.v.), a gram glutamine suspended in 10 ml solution of ml 0.9% daily for seven consecutive days, and injected with cisplatin (20 mg/kg I.p) on the seventh day to induce nephrotoxicity (P2). All groups received equivalent volumes of the used vehicles. Mice were sacrificed on the tenth day. The longitudinal section of the left kidney was excised from each animal for immunohistochemical examination.

Glutamine product was purchased from Serva, Germany, cisplatin product from Kalbe Farma, POD apoptosis detection kit (11684817910) from ROCHE, and anti-AIF antibody (AIF monoclonal antibody) from ThermoFisher Scientific (Cat. # MA5-15880).

### 2.2. Immunohistochemical Examination

Kidney tissue samples were fixed in 10% buffered neutral formalin, embedded in paraffin, cut, and stained with immunohistochemical staining for AIF expression and apoptosis examination using light microscopy.

Immunohistochemical detection of AIF expression was conducted using anti-AIF antibodies (AIF monoclonal antibody, ThermoFisher Scientific, Cat. # MA5-15880). Immunohistochemical staining was carried out with anti-AIF antibodies in brief steps as follows: deparaffinated preparations were on glass objects, washed with PBS pH 7.4, blocking endogenous peroxide with 3% H_2_O_2_ for 20 minutes, blocking unspecific protein with 5% FBS, incubating with primary antibody (anti-AIF antibody) overnight at 4°C, incubating with conjugated anti-mouse biotin for 1 hour at room temperature, incubating with Strep-avidin horseradish peroxidase for 40 minutes, drop DAB and incubating for 10 minutes. Counterstaining with Mayer hematoxylin, the preparation is rinsed with dH_2_O and aerated, mounted with a swab, and the preparation is covered with a glass cover.

Immunohistochemical detection of apoptosis was processing by apoptotic detection kit, POD (11684817910, ROCHE), with the following brief steps: deparaffinated tissue, preparations were given proteinase K for 15 minutes and dH_2_O in a Coplin jar for 2 × 2 minutes, removing endogenous peroxide with 3% H_2_O_2_ for 5 minutes at room temperature, drop the working strength of tdT enzyme in tissues, incubate at 37°C for 1 hour, place the preparation in a Coplin jar containing the working strength of the stop/wash buffer and incubated for 10 minutes at room temperature, drop antidigoxigenin conjugate incubation at room temperature in a damp container for 30 minutes, stain with substrate peroxidase for 10 minutes at room temperature, counterstaining with methyl green for 30 seconds at room temperature, and cover with a glass cover.

### 2.3. Statistical Analysis

The results of the AIF expression and apoptosis study used IHC staining and assessed with the H score show a significant difference in each group. The formula for calculating the H score is as follows: TS (total score) = PS (proportion score) + IS (intensity score) [14]. The data are expressed as means ± SEM. Statistical analysis was performed by one-way ANOVA followed by the LSD postanalysis test for multiple comparisons with *α* = 0.05, being considered as statistically significant, and Pearson correlation with *α* = 0.05, being considered as statistically significant.

## 3. Result

**Figure 1:**
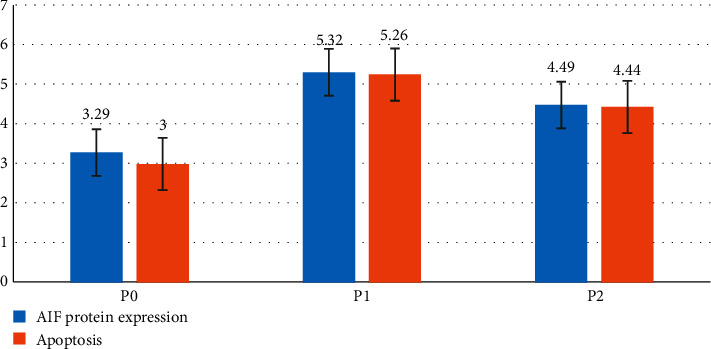
Graph of the mean score of AIF protein expression and apoptosis.

The results of the AIF expression and apoptosis study used IHC staining and assessed with the H score show a significant difference in each group (Figure 1). Administration of glutamine i.v before cisplatin i.p administration (P2) significantly decreased the amount of AIF expression compared to giving cisplatin without glutamine administration (P1). The mean score of AIF expression in P2 is 4.49 ± 0.47 compared to P1 with score 5.32 ± 0.68 (Tables 1–3). Administration of glutamine i.v before cisplatin i.p administration (P2) significantly decreased the amount of AIF expression compared to giving cisplatin without glutamine administration (P1) (Tables 1–5).

ANOVA test results on the AIF expression variable (Table 4) showed different evidence between groups in 1 research variable. With the LSD comparison test between P0 and P1, the mean score of AIF expression P0 = 3.29 ± 0.79 and P1 = 5.32 ± 0.68 (Tables 1 and 5) had a significant difference from the expression of AIF protein in the glomerular visceral epithelial cells (podocyte). LSD test results on P1 and P2 with a mean score of P1 = 5.32 ± 0.68 and P2 = 4.49 ± 0.47 (Table 5) had a decreased AIF excretion involved in glomerular visceral epithelial cells (podocytes).

Administration of glutamine i.v before cisplatin i.p administration (P2) significantly decreased the amount of apoptosis compared with giving cisplatin without glutamine administration (P1) (Tables 1–5).

ANOVA test results on the apoptosis variable (Table 4) showed a significant difference between groups in 1 research variable. With the LSD double comparison test between P0 and P1 with a mean apoptosis score of P0 = 3.04 ± 0.70 and P1 = 5.26 ± 0.53 (Tables 1 and 5) had a significant difference from the number of cells experiencing apoptosis in the glomerular visceral epithelial cells (podocyte). LSD test results on P1 and P2 with an average score of P1 = 5.26 ± 0.53 and P2 = 4.44 ± 0.41 (Table 5) had a significant reduction in the number of apoptosis in glomerular visceral epithelial cells (podocytes).

The results of the Pearson correlation test on the AIF and apoptosis expression (Tables 6 and 7) variables showed that the correlation was very strong with the direction of the relationship being directly proportional.

## 4. Discussion

In this study, mice were divided into three groups such as the negative control group (P0), the positive control group (P1), and the treatment group (P2), and each group consisted of 10 male white mice. P1 had the largest mean AIF expression and apoptosis compared to other groups.

The research data were analyzed using the ANOVA test, and if there were differences, it would be followed by a multiple comparison test, LSD. To use the ANOVA test, several conditions must be fulfilled: the sample must come from independent data, the variance between groups must be homogeneous, and the data in each group must be normally distributed. The homogeneity test in this study used the Levene test and showed a significance value > 0.05, which means the data in this study were homogeneous; the normality test used the Shapiro–Wilk test and showed the significance value > 0.05, which means the data in this study were normally distributed (Tables 2–5).

The research data were analyzed using Pearson correlation. To use the Pearson correlation, the following condition must be fulfilled: the data must be normal. The normality test in this study used the Kolmogorov–Smirnov test and showed the significance value > 0.5, which means the data in this study were normal (Tables 6 and 7).

**Figure 2:**
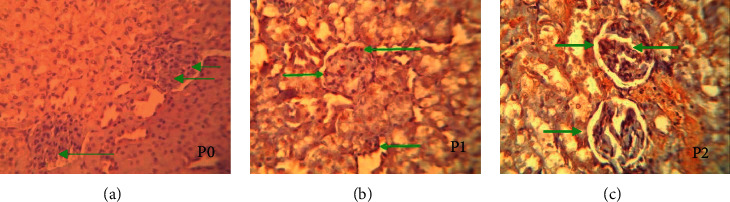
Morphology of the glomerular epithelial cells given anti-AIF antibodies. (a) P0 is the control, in which the mouse's kidney is not treated. (b) P1 is the kidney of mouse given cisplatin i.p on the 7th day. (c) P2 is the kidney of mouse injected with glutamine i.v 7 days in a row before being injected with cisplatin i.p on the 7th day. Positive if the cytoplasm and nucleus are colored brown.

**Figure 3:**
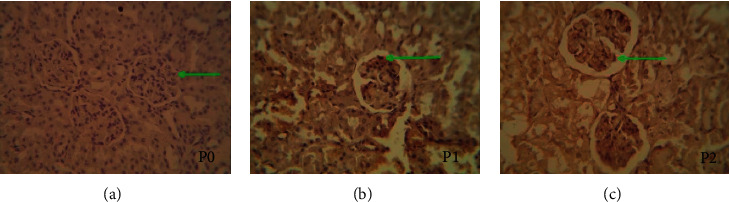
Morphology of the glomerular epithelial cells treated with apoptotic detection kit. (a) P0 is control, in which the mouse's kidney is not treated. (b) P1 is the kidney of mouse given cisplatin i.p on the 7th day. (c) P2 is the kidney of mouse injected with glutamine i.v 7 days in a row before being injected with cisplatin i.p on the 7th day. Positive if the cytoplasm and nucleus are colored brown.

Apoptosis and AIF excretion were analyzed in glomerular visceral epithelial cells (podocyte) in all groups in this study. Apoptosis and AIF expression in the positive control groups (P1) and (P2) are apoptotic processes triggered by cisplatin chemotherapy as a substance that has a nephrotoxic effect (Figures 2 and 3). Cisplatin-induced nephrotoxicity can occur from several pathways, including extrinsic apoptosis and intrinsic apoptosis that can trigger AIF expression, cell regulators, MAPK, inflammation, and ROS. Meanwhile, apoptosis in the negative control group (P0) can be caused by physiological processes that can be experienced by all cells. The influence of external variables that cannot be controlled can also cause apoptosis in the glomerular visceral epithelial cells (podocyte).

**Figure 4:**
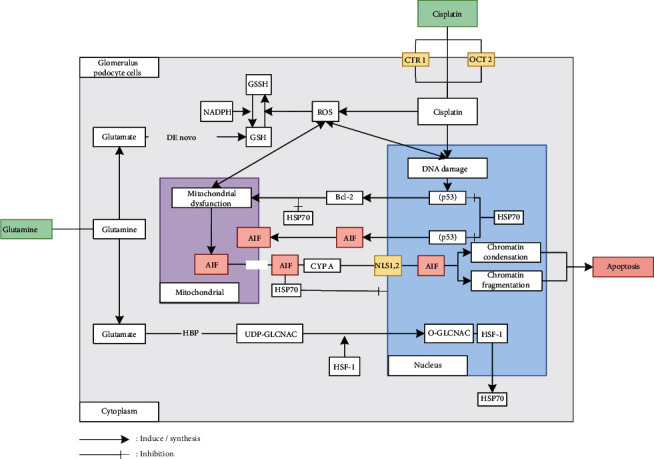
Conceptual framework.

The increase in AIF excretion in the positive control group (P1) was caused by exposure to cisplatin, an increase in free radicals and DNA damage which caused the maturation of the p53 gene to induce AIF protein transcription and induce Bcl-2 causing mitochondrial dysfunction to form holes in the mitochondrial membrane, so that the AIF protein could translocate to the cytoplasm and nucleus (Figure 4).

These results are consistent with previous studies conducted by [15], which stated that translocation of AIF protein from mitochondria can be induced by cisplatin in chemosensitive ovarian cancer cells and causes apoptosis. Apoptosis, which represents the form of cell death performed by caspases, has traditionally been the only form of physiological and programmed cell death. However, recent evidence suggests that programmed cell death (PCD) can occur in the absence of caspase activation at all. Indeed, a large number of caspase-independent models are now defined, and a key protein involved in this type of PCD, the apoptosis-inducing factor (AIF), has been identified. Cisplatin causes changes in the mitochondrial PTP to cause the pores in the mitochondria to open and allow small molecules such as the AIF protein to escape [15].

The decrease in AIF expression in the treatment group (P2) was due to glutamine, which is the precursor to glutathione in cells, directly binding to free radicals which can prevent mitochondrial dysfunction due to cisplatin administration. In addition, glutamine can increase the expression of Hsp70 which is an antiapoptotic agent that can prevent various cell death pathways, one of which is by inhibiting the maturation of p53 to induce Bcl-2, preventing Bcl-2 from causing mitochondrial dysfunction, so that translocation of AIF protein from mitochondria can be prevented (Figure 4).

These results are consistent with previous studies conducted by [16], showing that HSP70 modulates apoptosis of the caspase-independent pathway in primary cortical neurons and SH-SY5Y cells through interaction with AIF and by preventing translocation to the nucleus [16].

The increase in apoptosis in the positive control group (P1) due to cisplatin exposure triggered an increase in free radicals that can activate various cell death pathways. In addition, cisplatin induces maturation of the p53 gene and induces apoptosis (Figure 4).

These results are consistent with previous studies conducted by [17], showing that cisplatin exposure induces a mitochondrial-dependent ROS response that significantly contributes to cell killing by enhancing the cytotoxic effect exerted through the formation of nDNA damage [15], which states that translocation of the AIF protein from mitochondria can be induced by cisplatin in chemosensitive ovarian cancer cells and causes apoptosis. [15, 17].

The decrease in apoptosis in the treatment group (P2) was due to glutamine which is a precursor to GSH, which is a powerful antioxidant that can bind free radicals triggered by cisplatin administration. In addition, glutamine can increase the expression of Hsp70 which is an antiapoptotic agent by preventing apoptosis of the caspase-independent pathway and the caspase-dependent pathway, so that apoptosis due to cisplatin can be prevented (Figure 4).

These results are in accordance with the theory described by [12, 18–20] who stated that glutamine is a precursor of GSH where GSH is a powerful antioxidant and plays an important role in the metabolism of exogenous and endogenous substances. GSH participates in many cellular reactions. It directly scavenges free radicals and other reactive oxygen species (hydroxyl radicals, lipid peroxyl radicals, peroxynitrite, and H2O2) and is indirectly linked to enzymatic reactions that can decrease apoptosis. In addition, glutamine can increase the expression of Hsp70 which is an antiapoptotic agent through the HBP pathway and can also reduce apoptosis [12, 18–20].

Increased AIF expression and increased apoptosis in the positive control group (P1) were strongly associated. The increase in AIF expression caused by cisplatin exposure triggers AIF synthesis and AIF translocation to the nucleus and causes condensation and large-scale chromatin fragmentation which triggers apoptosis (Figure 4).

These results are consistent with previous studies conducted by [15], which stated that translocation of AIF protein from mitochondria can be induced by cisplatin in chemosensitive ovarian cancer cells and causes apoptosis [15].

The decrease in AIF expression and the decrease in apoptosis in the treatment group (P2) were strongly associated. The decrease in AIF expression in the group injected with glutamine prior to cisplatin administration was due to the nephroprotective effect of glutamine as an antioxidant precursor that can bind to antioxidants directly, and so, it can inhibit/prevent mitochondrial dysfunction which can cause AIF protein to translocate to the cytoplasm and nucleus. In addition, glutamine can increase the expression of Hsp70 which is an antiapoptotic agent by preventing translocation of AIF to the nucleus, maturation of the p53 gene, and Bcl-2 proapoptosis, prevents AIF protein from translocating to the cytoplasm and nucleus, and causes large-scale condensation and fragmentation of chromatin, thus preventing apoptosis (Figure 2).

These results are in accordance with previous studies which stated that Hsp72 inhibits the release of AIF protein from mitochondria which can inhibit apoptosis in renal epithelial cells exposed to metabolic inhibitors [21].

## 5. Conclusion

Based on the research that has been conducted, there is a change in the expression of AIF and apoptotic cells in intravenous glutamine administration on glomerular visceral epithelial cells (podocyte) of male white mice exposed to cisplatin; the changes that occur in this study are in the form of a decrease. Decreased expression of AIF on intravenous glutamine administration is the correlation with a decrease in apoptosis in the glomerular visceral cells (podocyte). Glutamine administration can decrease AIF expression and apoptosis induced by cisplatin administration. Glutamine administration might represent the treatment of nephrotoxic-induced cisplatin.

## Figures and Tables

**Table 1:** Descriptive statistic of the AIF protein expression and apoptotic cells.

Variable	AIF protein expression	Apoptotic cells
	Average ± SD	Average ± SD
P0	3.29 ± 0.79	3.04 ± 0.69
P1	5.32 ± 0.68	5.26 ± 0.53
P2	4.49 ± 0.47	4.44 ± 0.41

P0, control, in which the mouse's kidney is not treated; P1, the kidney of mouse given cisplatin i.p; P2, the kidney of mouse injected with glutamine i.v on the 7th day.

**Table 2:** The result of the normality using the Shapiro–Wilk test.

Variable	AIF protein expression	Apoptotic cells
	Significance	Significance
P0	0.702	0.137
P1	0.539	0.189
P2	0.733	0.647

Significance value > 0.05 means the data in this study are normally distributed.

**Table 3:** The result of homogeneity variances using Levene' statistic test.

Variable	AIF protein expression	Apoptotic cells
	Significance	Significance
Levene statistic	0.150	0.448

Significance value > 0.05 means the data in this study are homogeneous.

**Table 4:** The result of ANOVA.

Variable	AIF protein expression	Apoptotic cells
	Significance	Significance
ANOVA	0.000	0.000

Significance value > 0.05 means the data in this study had different results.

**Table 5:** The result of LSD.

Variable	Comparison		Significance	Interpretation
AIF protein expression	P0	P1	0.000	Obtained difference
		P2	0.000	
	P1	P2	0.009	

Apoptotic cells	P0	P1	0.000	Obtained difference
		P2	0.000	
	P1	P2	0.003	

Significance value > 0.05 means the data in this study had different results in each group.

**Table 6:** The result of normality test using the Kolmogorov–Smirnov test.

Variable	AIF protein expression	Apoptotic cells
	Significance (2-tailed)	Significance (2-tailed)
Kolmogorov–Smirnov test	0.200	0.200

Significance value > 0.05 means the data in this study are normally distributed.

**Table 7:** The result of Pearson correlation.

Variable	Correlation	Significance	Interpretation
AIF protein expression and apoptotic cells	0.928	0.00	Correlation is significant and linear

*r* (Pearson correlation) > 0.349 (positive) means the data in this study had a correlation significant and linear. Thus, the significance value > 0.05 means the data in this study had a significant correlation between AIF protein expression and apoptosis cells.

## Data Availability

The data used to support the findings of this study are included within the article and are also available online.

## Additional Points

The main sample, male Wistar rats, in this research was collected from Unit Pemeliharaan Hewan Coba Medical Pharmacology Department of Universitas Airlangaa. The number or size of the Wistar rat sample was determined using the Federer formula. The number of Wistar rats is 30. The kidney samples were collected by sacrificing the rats on day 10 to measure AIF expression and doing apoptosis examination.The data were processed using statistical software SPSS version 17. Parametric statistical tests are used if the data curve is normally distributed; if the data curve is slanted, nonparametric statistical tests will be used. The data show that there is a decreased expression of AIF on intravenous glutamine administration, followed by a decrease in apoptosis in the podocyte.
